# Polymicrobial intensive care unit-acquired pneumonia: prevalence, microbiology and outcome

**DOI:** 10.1186/s13054-015-1165-5

**Published:** 2015-12-10

**Authors:** Miquel Ferrer, Leonardo Filippo Difrancesco, Adamantia Liapikou, Mariano Rinaudo, Marco Carbonara, Gianluigi Li Bassi, Albert Gabarrus, Antoni Torres

**Affiliations:** 1 Department of Pneumology, Thorax Institute, Hospital Clinic, Villarroel 170, 08036 Barcelona, Spain; 2 Institut d’Investigacions Biomèdiques August Pi I Sunyer (IDIBAPS), Barcelona, Spain; 3 Centro de Investigación Biomédica en Red-Enfermedades Respiratorias (CibeRes CB06/06/0028)-Instituto de Salud Carlos III (ISCiii), Madrid, Spain; 4 Department of Internal Medicine, Ospedale Sant’Andrea, “Sapienza” University, Via di Grottarossa 1035-1039, Rome, Italy; 5 Sotiria Chest Diseases Hospital, 6rd Respiratory Department, Mesogion 152, Athens, Greece; 6 Department of Anesthesia, Università degli Studi di Milano, IRCCS Fondazione Ospedale Maggiore Policlinico Cà Granda Milano, Milan, Italy

**Keywords:** Hospital-acquired pneumonia, ICU-acquired pneumonia, Ventilator-acquired pneumonia, Polymicrobial pneumonia

## Abstract

**Background:**

Microbial aetiology of intensive care unit (ICU)-acquired pneumonia (ICUAP) determines antibiotic treatment and outcomes. The impact of polymicrobial ICUAP is not extensively known. We therefore investigated the characteristics and outcomes of polymicrobial aetiology of ICUAP.

**Method:**

Patients with ICUAP confirmed microbiologically were prospectively compared according to identification of 1 (monomicrobial) or more (polymicrobial) potentially-pathogenic microorganisms. Microbes usually considered as non-pathogenic were not considered for the etiologic diagnosis. We assessed clinical characteristics, microbiology, inflammatory biomarkers and outcome variables.

**Results:**

Among 441 consecutive patients with ICUAP, 256 (58 %) had microbiologic confirmation, and 41 (16 %) of them polymicrobial pneumonia. Methicillin-sensitive *Staphylococcus aureus*, *Haemophilus influenzae*, and several *Enterobacteriaceae* were more frequent in polymicrobial pneumonia. Multi-drug and extensive-drug resistance was similarly frequent in both groups. Compared with monomicrobial, patients with polymicrobial pneumonia had less frequently chronic heart disease (6, 15 % vs*.* 71, 33 %, p = 0.019), and more frequently pleural effusion (18, 50 %, vs*.* 54, 25 %, p = 0.008), without any other significant difference. Appropriate empiric antimicrobial treatment was similarly frequent in the monomicrobial (185, 86 %) and the polymicrobial group (39, 95 %), as were the initial response to the empiric treatment, length of stay and mortality. Systemic inflammatory response was similar comparing monomicrobial with polymicrobial ICUAP.

**Conclusion:**

The aetiology of ICUAP confirmed microbiologically was polymicrobial in 16 % cases. Pleural effusion and absence of chronic heart disease are associated with polymicrobial pneumonia. When empiric treatment is frequently appropriate, polymicrobial aetiology does not influence the outcome of ICUAP.

## Background

Intensive care unit (ICU)-acquired pneumonia (ICUAP) is the leading infection in critically-ill patients, accounting for prolonged mechanical ventilation and length of stay, and poor outcome [1–4]. The use of inappropriate initial antibiotic therapy is a major determinant of mortality in patients with ICUAP [5], emphasizing the importance of a timely and accurate therapy for this infection [6]. For this reason, it is often necessary to use a combination of broad-spectrum empiric antibiotics, particularly in patients who are at risk for difficult-to-treat bacteria [7, 8]. Recent investigations have shown that multi-drug-resistant (MDR) or high-risk pathogens have been isolated in around half of patients with an episode of ventilator-associated pneumonia (VAP) or ICUAP confirmed microbiologically [9, 10].

ICUAP, and particularly VAP, can be caused by more than one microbial pathogen. Multiple etiologic pathogens are potentially an additional challenge for achieving appropriate antimicrobial treatment in these patients. A previous study reported a 48 % rate of polymicrobial etiology in episodes of VAP with microbiologic confirmation [11]. These authors concluded that the epidemiology and outcomes of patients with monomicrobial and polymicrobial VAP did not differ significantly. However, in this study, a substantial proportion of episodes classified as polymicrobial VAP had positive isolation of bacteria usually considered as non-pathogenic microorganisms. Moreover, between 15 % and 73 % patients with an episode of ICUAP are not previously intubated [2, 12, 13], namely non-ventilator ICUAP (NV-ICUAP).

To our knowledge, no previous studies have comprehensively assessed polymicrobial ICUAP strictly considering the identification of potentially pathogenic microorganisms (PPM). We have recently shown that positive microbiology is associated with worse outcomes in patients with clinical diagnosis of ICUAP [14]. Therefore, whether patients with polymicrobial etiology of ICUAP have different characteristics and outcomes to those with monomicrobial etiology is unknown. We therefore investigated the incidence, characteristics, risk factors, systemic inflammatory response and outcomes of polymicrobial, compared with monomicrobial, etiology of ICUAP.

## Methods

### Study population

The study was conducted between October 2004 and September 2013 in six medical and surgical ICUs, comprising 45 beds, at Hospital Clinic, Barcelona, Spain, an 800-bed university hospital. The investigators made daily rounds in each ICU. Patients older than 18 years, admitted to these ICUs for 48 h or more, with clinical diagnosis of ICUAP were consecutively enrolled in the study, and this being only the first episode, were analyzed. Exclusion criteria were: 1) severe immune suppression (neutropenia after chemotherapy or hematopoietic transplant, drug-induced immune suppression in solid-organ transplant or cytotoxic therapy, and patients with human immunodeficiency virus) and 2) absence of microbiologic confirmation. The institution’s Internal Review Board approved the study (Comite Etic d’Investigacio Clinica, registry number 2009/5427) and written informed consent was obtained from patients or their next of kin.

### Definition of pneumonia, microbiologic processing, and antimicrobial treatment

Clinical diagnosis of ICUAP was based on clinical criteria: new or progressive radiological pulmonary infiltrate together with at least two of the following: temperature >38 °C or <36 °C, leukocytosis >12,000/mm^3^ or leukopenia <4,000/mm^3^, and purulent respiratory secretions [1, 15, 16]. Non-ventilated ICUAP was defined when patients acquired pneumonia after more than 48 h of ICU admission and without previous mechanical ventilation [2]. We considered VAP in patients with previous invasive mechanical ventilation for 48 h or more. Early-onset pneumonia was defined as occurring within the first 4 days of hospitalization [1].

The microbiologic evaluation included the collection of at least one lower respiratory tract sample: sputum in non-ventilated patients, tracheobronchial aspirates (TBAS) in intubated patients, and/or bronchoscopic [17] or blind bronchoalveolar lavage (BAL) [18], whenever possible, within the first 24 h of inclusion [19]. Bronchoscopic BAL and blind BAL were performed as previously described [19].

The same sampling method was performed on the third day if clinically indicated. Blood cultures and cultures from pleural fluid, if puncture was indicated, were also taken. Urinary antigens of *Legionella pneumophila* and *Streptococcus pneumoniae* were systematically collected. Microbiologic confirmation of pneumonia was defined by the presence of at least one PPM in respiratory samples above predefined thresholds (BAL >10^4^, sputum or TBAS >10^5^ colony-forming units/mL, respectively), in pleural fluid or in blood cultures if an alternative cause of bacteremia was ruled out [20, 21].

Drug resistance of pathogens was defined according to a recent report [22]. MDR pathogens were defined as acquired non-susceptibility to at least one agent in three or more antimicrobial categories. Extensive drug resistance (XDR) was defined as non-susceptibility to at least one agent in all but two or fewer antimicrobial categories (i.e., bacterial isolates remain susceptible to only one or two categories). Pan drug resistance (PDR) was defined as non-susceptibility to all agents in all antimicrobial categories. We considered methicillin-resistant *Staphylococcus aureus* (MRSA), and *Enterobacteriaceae* producing extended-spectrum β-lactamase as MDR pathogens [9].

Monomicrobial and polymicrobial pneumonia were defined when one and more than one PPM, respectively, were identified as etiologic agents at onset of pneumonia. Isolation of *Candida* spp, *Streptococcus viridans*, *Staphylococcus epidermidis*, *Neisseria* spp, *Enterococcus* spp, and *Corynebacterium* spp in lower respiratory tract samples were not considered etiologic agents.

The initial empiric antimicrobial treatment was administered according to local adaptation of the American Thoracic Society/Infectious Disease Society of America guidelines [1], based on the most frequently isolated PPM and their patterns of antimicrobial sensitivity in our institution, and subsequently revised according to the microbiologic results. The empirical antimicrobial treatment was considered appropriate when the isolated pathogens were susceptible in vitro to at least one of the antimicrobials administered at an adequate dose [8].

We assessed the initial response to treatment after 72 to 96 h of antimicrobial treatment, as previously described [23, 24]. Non-response was considered when at least one of the following criteria were present: 1) no improvement of the arterial O_2_ tension to inspired O_2_ fraction ratio or need for intubation because of pneumonia (defined as need for intubation after 24 h from the beginning of antibiotics); 2) persistence of fever (temperature ≥38 °C) or hypothermia (<35.5 °C) together with purulent respiratory secretions; 3) increase in the pulmonary infiltrates on chest radiograph ≥50 %; or 4) occurrence of septic shock or multiple organ dysfunction syndrome, defined as three or more organ system failures not present on day 1.

### Assessment of systemic inflammatory response

We evaluated the serum levels of interleukin (IL)-6, IL-8, tumor necrosis factor-alpha (TNF-alpha), C-reactive protein, procalcitonin and mid-regional pro-adrenomedullin within the first 24 h after the diagnosis of pneumonia. All methods of these analyses have been recently described in detail [25, 26].

### Data collection

All relevant data were collected at admission and at onset of pneumonia from the medical records and bedside flow charts, including laboratory, radiologic and microbiologic information. We calculated the acute physiology and chronic health evaluation (APACHE)-II score [27] and the simplified acute physiology score (SAPS)-II [28] on ICU admission. The simplified clinical pulmonary infectious score (CPIS) [29] and the sepsis-related organ failure assessment (SOFA) [30] scores were also evaluated on ICU admission and up to 9 days after the onset of pneumonia. Septic shock [31] and acute respiratory distress syndrome (ARDS) [32] were defined according to previously described criteria. Patients were followed until death or up to 90 days after the diagnosis of pneumonia.

### Outcome variables

The outcomes of patients with monomicrobial pneumonia were compared to those with polymicrobial pneumonia. The primary outcome variable was mortality at 90 days after the diagnosis of ICUAP. Secondary outcomes included initial non-response to treatment, length of ICU and hospital stay, ventilator-free days at day 28 [33], and mortality at 28 days.

### Statistical analysis

Categorical and continuous data are presented as number (percentage) and as mean ± SD (or median (inter-quartile range)), respectively. Categorical variables were compared with the chi square (*χ* ^2^) test or the Fisher exact test. Quantitative continuous variables were compared using the *t* test or the Mann–Whitney test for normally and non-normally distributed variables, respectively. Survival curves for patients with monomicrobial and polymicrobial pneumonia were obtained using the Kaplan-Meier method and compared using the log-rank test.

The association between polymicrobial or monomicrobial etiology and patients’ outcomes was adjusted for variables potentially related to mortality, such as age, APACHE-II and SAPS scores at ICU admission, SOFA score, CPIS and arterial partial pressure of oxygen/inspired oxygen fraction (PaO_2_/FiO_2_) ratio at onset of pneumonia, VAP or NV-ICUAP, and unilateral or bilateral chest x-ray infiltrates. We used Cox proportional hazard regression analysis for 28-day and 90-day mortality.

All reported *P* values are two-sided and not adjusted for multiple comparisons. A *P* value <0.05 was considered significant. All statistical analyses were performed using IBM SPSS Statistics Version 20 (Armonk, NY, USA).

## Results

### Patients’ characteristics

**Fig. 1:**
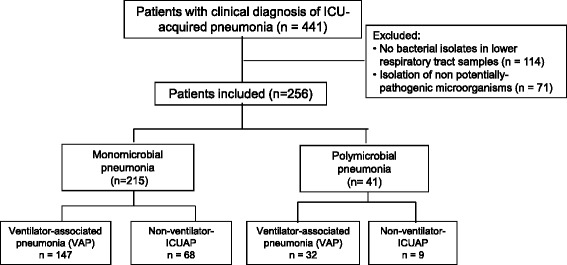
Trial profile of included and excluded patients. *ICU* intensive care unit, *VAP* ventilator-associated pneumonia, *ICUAP* ICU-acquired pneumonia

We prospectively identified 441 consecutive patients with ICUAP; 185 (42 %) were excluded because a positive microbiological diagnosis could not be made. Therefore, we included 256 patients: 215 (84 %) with monomicrobial, and 41 (16 %) with polymicrobial ICUAP (Fig. 1).

The characteristics of patients at ICU admission and at onset of pneumonia are summarized in Tables 1 and 2. The rate of chronic heart disease was lower, and the rate of pleural effusion was higher, in patients with polymicrobial, compared to monomicrobial pneumonia. No other significant differences were found between the two groups in the remaining baseline characteristics, co-morbidities, reasons for ICU admission, disease severity, and laboratory variables at the onset of pneumonia.

**Table 1:** Baseline characteristics of patients at ICU admission

	Monomicrobial pneumonia n = 215	Polymicrobial pneumonia n = 41	*P* value
Age, years	63 ± 16	60 ± 17	0.34
Sex, male, n (%)	155 (72)	31 (76)	0.64
APACHE-II score	17 ± 6	18 ± 6	0.52
SAPS-II score	41 ± 15	42 ± 13	0.66
SOFA score	7.2 ± 3.1	7.8 ± 3.3	0.28
Current or former smokers, n (%)	113 (53)	20 (49)	0.66
Current or former alcohol abuse, n (%)	57 (27)	10 (24)	0.77
Co-morbidities, n (%)			
Diabetes mellitus	47 (22)	10 (24)	0.72
Chronic renal failure	17 (8)	2 (5)	0.50
Solid cancer	41 (19)	3 (7)	0.068
Chronic heart disorders	71 (33)	6 (15)	0.019
Chronic lung disease	74 (34)	10 (24)	0.21
Chronic liver disease	36 (17)	8 (20)	0.67
Recent surgery, n (%)	97 (45)	15 (37)	0.31
Tracheostomy at admission, n (%)	24 (11)	4 (10)	0.78
Main causes of ICU admission, n (%)			
Postoperative	38 (18)	4 (10)	0.31
Hypoxemic respiratory failure	37 (17)	7 (17)	0.85
Decreased consciousness	31 (14)	8 (20)	0.56
Hypercapnic respiratory failure	26 (12)	2 (5)	0.28
Cardiac arrest	15 (7)	4 (10)	0.77
Septic shock	17 (8)	3 (7)	0.85
Multiple trauma	18 (8)	6 (15)	0.34
Non-surgical abdominal disease	10 (5)	3 (7)	0.75
Previous antibiotics in the ICU, n (%)	163 (76)	31 (76)	0.98
Previous cardiopulmonary resuscitation, n (%)	23 (11)	4 (10)	0.86

*APACHE* acute physiology and chronic health evaluation; *SAPS* simplified acute physiology score; *SOFA* sepsis-related organ failure assessment; *ICU* intensive care unit

**Table 2:** Characteristics of patients at onset of pneumonia

	Monomicrobial pneumonia n = 215	Polymicrobial pneumonia n = 41	*P* value
Ventilator-associated pneumonia, n (%)	147 (68)	32 (78)	0.22
Non-ventilator-ICUAP, n (%)	68 (32)	9 (22)	
Early-onset pneumonia, n (%)	55 (26)	14 (34)	0.27
Late-onset pneumonia, n (%)	159 (74)	27 (66)	
ICU stay before pneumonia, days	7 ± 8	8 ± 9	0.79
Hospital stay before pneumonia, days	12 ± 13	10 ± 9	0.26
SOFA score	7.5 ± 3.4	7.9 ± 3.0	0.51
CPIS day 1	6.4 ± 1.5	6.9 ± 1.5	0.026
Previous use of corticosteroids, n (%)	81 (38)	20 (50)	0.14
Previous airway colonization, n (%)	87 (41)	12 (29)	0.18
ARDS criteria, n (%)	22 (10)	7 (18)	0.16
Pleural effusion, n (%)	52 (25)	20 (49)	0.002
Shock at onset of pneumonia, n (%)	101 (47)	19 (46)	0.94
Serum creatinine, mg/Dl	1.25 ± 1.04	1.15 ± 0.90	0.57
Blood hemoglobin, g/L	10.5 ± 1.7	10.9 ± 1.9	0.14
White blood cell count, L^−9^	14,068 ± 7,282	12,510 ± 4,893	0.19
PaO_2_/FiO_2_, mmHg	210 ± 82	202 ± 80	0.56

*ICU* intensive care uni, *ICUAP* ICU-acquired pneumonia, *SOFA* sepsis-related organ failure assessment, *CPIS* clinical pulmonary infection score, *ARDS* acute respiratory distress syndrome, *PaO*_*2*_*/FiO*_*2*_ ratio of arterial oxygen tension to inspired oxygen fraction

### Microbiologic assessment

The proportions of lower respiratory tract samples processed for microbiology were similar in the two groups (Table 3). However, blood and pleural fluid culture were performed more often in patients with polymicrobial pneumonia, in case of pleural fluid, owing to the higher incidence of pleural effusion in this group.

**Table 3:** Diagnostic samples processed for microbiologic culture

	Monomicrobial pneumonia n = 215	Polymicrobial pneumonia n = 41	*P* value
Any lower respiratory tract sample, n (%)	213 (99)	41 (100)	0.54
Tracheal aspirate or sputum, n (%)^a^	202 (94)	38 (93)	0.76
Bronchoalveolar lavage, n (%)	41 (19)	9 (22)	0.68
Pleural fluid culture, n (%)	18 (8)	9 (22)	0.009
Blood culture, n (%)	143 (67)	34 (83)	0.037

^a^Sputum or tracheal aspirates were obtained depending on whether or not patients were intubated at onset of pneumonia. In some patients, a sample of both sputum and tracheal aspirate were processed for culture

There were 2 patients (5 %) in the polymicrobial pneumonia group with methicillin-sensitive *Staphylococcus aureus* (MSSA) isolated in pleural fluid. In the monomicrobial pneumonia group, 8 patients (4 %) had positive pleural fluid cultures: MSSA and *Pseudomonas aeruginosa* in 3 patients, and MRSA in 2 patients.

There were 5 patients (12 %) in the polymicrobial pneumonia group with positive blood cultures: *Serratia* spp. in 2 patients, and MSSA, *Escherichia coli*, and *Fusobacterium* spp. in 1 patient. The pathogens concomitantly isolated in respiratory samples from these patients with bacteremia were: *Serratia* spp. (*Morganella morganii* and MSSA), MSSA (*S. pneumoniae*), *E. coli* (MSSA), and *Fusobacterium* spp. (*E. coli*). In the monomicrobial pneumonia group, 18 patients (8 %) had positive blood cultures: *P. aeruginosa* in 4 patients, MRSA and *E. coli* in 3 patients, MSSA and *Klebsiella* spp. in 2 patients, and *Stenotrophomonas maltophilia*, *Citrobacter* spp., *Bacteroides fragilis* and *Proteus mirabilis* in 1 patients.

The etiologic diagnosis of pneumonia is shown in Table 4. All patients with polymicrobial pneumonia had two different pathogens identified, except two who had three different pathogens identified (Table 5). The most frequently isolated pathogens were *P. aeruginosa*, *Enterobacteriaceae*, and MSSA. Several bacteria, such as MSSA, *Haemophilus influenzae*, *Klebsiella* spp., *E. coli*, *Enterobacter* spp., *Citrobacter* spp., and *Serratia* spp., were more frequently isolated in patients with polymicrobial pneumonia. The remaining pathogens were isolated at similar rates in both groups. These findings were similar when we analyzed patients with VAP and non-ventilator ICUAP separately.

**Table 4:** Etiologic diagnosis of pneumonia

Pathogen, n (%)	Monomicrobial pneumonia n = 215	Polymicrobial pneumonia n = 41	*P* value
Gram-positive bacteria			
Methicillin-sensitive *Staphylococcus aureus*	39 (18)	14 (34)	0.037
Methicillin-resistant *Staphylococcus aureus*	18 (8)	3 (7)	0.94
*Streptococcus pneumoniae*	10 (5)	3 (7)	0.75
Gram-negative bacteria			
*Enterobacteriaceae*			
*Klebsiella* spp.	14 (7)	9 (22)	0.005
*Escherichia coli*	9 (4)	10 (24)	<0.001
*Proteus* spp.	3 (1)	3 (7)	0.087
*Enterobacter* spp.	9 (4)	6 (15)	0.025
*Citrobacter* spp.	2 (1)	3 (7)	0.039
*Serratia* spp.	9 (4)	6 (15)	0.025
*Morganella morganii*	1 (0.5)	1 (2.4)	0.73
*Haemophilus influenza*	6 (3)	5 (12)	0.023
Non-fermenting Gram-negative bacilli			
*Stenotrophomonas maltophilia*	12 (6)	4 (10)	0.51
*Pseudomonas aeruginosa*	74 (34)	12 (29)	0.65
*Acinetobacter* spp.	0	1 (2.4)	0.38
*Moraxella catarrhalis*	2 (0.9)	1 (2.4)	0.98
*Fusobacterium* spp.	0	1 (2.4)	0.38
*Bacteroides fragilis*	1 (0.5)	0	0.36
Fungi			
*Aspergillus* spp.	6 (3)	2 (5)	0.84
Bacteremia	18 (8)	5 (12)	0.63
Patients with drug-resistant pathogens:			0.61
MDR pathogens	38 (18)	9 (22)	
XDR pathogens	19 (9)	2 (5)	
MDR pathogens isolated^a^:			
Methicillin-resistant *S. aureus*	18	3	
Non-fermenting Gram-negative bacilli	12	1	
*Enterobacteriaceae*	8	7	
XDR pathogens isolated:			
Non-fermenting Gram-negative bacilli	18	2	
*Enterobacteriaceae*	1	0	
Microbiologic evolution on day 3:			
Persistence of 2 pathogens from day 1	-	5 (12)	
Persistence of 1 pathogen from day 1	74 (34)	13 (32)	
Persistence + new pathogen isolated	12 (6)	0 (0)	
Eradication but new pathogen isolated	12 (6)	1 (2)	
Eradication of initial pathogen	71 (33)	14 (34)	
Microbiologic evaluation on day 3 not done	46 (21)	8 (20)	

^a^In two patients with polymicrobial pneumonia, both pathogens were multi-drug-resistant (*MDR*). *XDR* extensive-drug-resistant

**Table 5:** Combinations of pathogens according to microbial groups in patients with polymicrobial pneumonia

Pathogens	Number
Patients with 3 pathogens identified	
*Enterobacteriaceae* + MSSA + non-fermenting GNB	1
*Enterobacteriaceae* + MSSA + *H. influenzae*	1
Patients with 2 pathogens identified	
2 *Enterobacteriaceae*	9
*Enterobacteriaceae* + MSSA	6
*Enterobacteriaceae* + non-fermenting GNB	6
2 non-fermenting GNB	3
*Enterobacteriaceae* + *H. influenzae*	2
*Enterobacteriaceae* + MRSA	2
MSSA + *S. pneumoniae*	2
MSSA + non-fermenting GNB	2
*Enterobacteriaceae* + *M. catarrhalis*	1
*Enterobacteriaceae* + *Fusobacterium* spp	1
MSSA + *H. influenza*	1
MSSA + *Aspergillus* spp.	1
Non-fermenting GNB + *Aspergillus* spp.	1
Non-fermenting GNB + MRSA	1
*S. pneumoniae* + *H. influenzae*	1

*MSSA* methicillin-sensitive *Staphylococcus aureus*, *GNB* Gram-negative bacilli, *MRSA* methicillin-resistant *Staphylococcus aureus*

The proportion of patients with MDR and XDR pathogens isolated were similar in both groups (Table 4). There were no patients with PDR microorganisms. Patients treated with antibiotics before the onset of ICUAP more frequently had MDR or XDR pathogens than those not treated previously with antibiotics (59 (30 %) vs*.* 9 (15 %), respectively, *p* = 0.014). A new microbiologic evaluation was done on the third day of evolution in 169 patients (79 %) and 33 patients (80 %) from the monomicrobial and polymicrobial groups, respectively. The microbiologic evolution is shown in Table 4.

### Assessment of systemic inflammatory response

The serum levels of all inflammatory biomarkers were similar in patients from the two groups (Table 6).

**Table 6:** Serum levels of inflammatory biomarkers

	N^a^	Monomicrobial pneumonia n = 215	N^a^	Polymicrobial pneumonia n = 41	*P* value
C-reactive protein, mg/dL	206	13 (7;22)	36	13 (4;25)	0.75
IL-6 day 1, pg/mL	107	157 (49:462)	19	92 (34;213)	0.19
IL-8 day 1, pg/mL	107	99 (59;209)	19	69 (55;161)	0.33
TNF-alpha day 1, pg/mL	107	8 (5;16)	19	7 (3;10)	0.30
Procalcitonin day 1, ng/mL	108	0.48 (0.15;1.57)	20	0.44 (0.06;1.72)	0.53
MR-proADM day 1, nmol/L	114	1.35 (0.55;2.28)	20	0.60 (0.25;2.02)	0.13

Reported values are median (interquartile range). ^a^Number of patients with samples processed for each inflammatory biomarker in each group. *IL* interleukin, *MR-proADM* mid-regional pro-adrenomedullin, *TNF* tumor necrosis factor

### Outcome variables

**Fig. 2:**
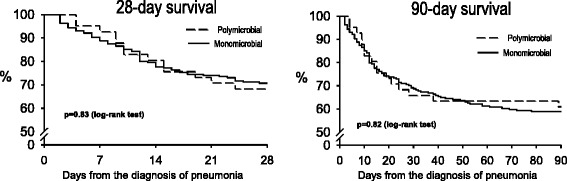
Kaplan-Meier survival curves at 28 and 90 days in the study cohort survivors in patients with monomicrobial and polymicrobial pneumonia

The appropriateness of the empirical antimicrobial treatment, the initial non-response to treatment, the length of stay, the ventilator-free days, and mortality at 28 and 90 days were similar in both groups (Table 7 and Fig. 2). Mortality of patients adjusted for initial non-response to treatment did not differ between groups (28 days: *p* = 0.71; 90 days: *p* = 0.49).

**Table 7:** Antimicrobial treatment, length of stay and outcomes

	Monomicrobial pneumonia n = 215	Polymicrobial pneumonia n = 41	*P* value
Appropriate empiric treatment, n (%)	185 (86)	39 (95)	0.11
Initial non-response to treatment, n (%)	115 (54)	28 (68)	0.080
ICU stay, days	23 ± 19	25 ± 20	0.47
Hospital stay, days	44 ± 34	43 ± 31	0.98
Ventilator-free days at day 28	11 (0;23)	0 (0;21)	0.26
Cause of death within 90 days, n (%)			0.26
Shock-multiple organ failure	65 (70)	9 (56)	
Refractory hypoxemia	11 (12)	2 (13)	
Order do-not-resuscitate	6 (7)	0	
Brain anoxia	8 (9)	4 (25)	
Other	2 (2)	1 (6)	

*ICU* intensive care unit

The most frequent cause of death was shock-multiple organ failure. Even when adjusted for variables potentially related to mortality, the polymicrobial etiology of ICUAP was not associated with 28-day mortality (adjusted hazard ratio 0.86, 95 % confidence interval 0.44–1.58, *p* = 0.65) or 90-day mortality (adjusted hazard ratio 1.16, 95 % confidence interval 0.57–2.39, *p* = 0.69).

## Discussion

Polymicrobial etiology accounted for 16 % cases of ICUAP with positive microbiology. Except for less frequent chronic heart disease and more frequent pleural effusion in polymicrobial pneumonia, there were no other significant differences between patients with monomicrobial and polymicrobial pneumonia in their baseline characteristics, inflammatory response or outcomes.

Information about the polymicrobial etiology of ICUAP is limited. The only study that specifically addressed this issue in VAP, published in 2002 [11], found a substantially higher proportion of polymicrobial etiology (48 %) compared to the present one. Unlike our study, those authors included some bacteria that are considered non-pathogenic for the lung in non-immunosuppressed patients, such as several *Streptococcus* species, *Neisseria* spp, *Enterococcus* spp, and coagulase-negative *Staphylococci*. Indeed, a substantial proportion of microbial isolates reported in polymicrobial VAP in that study (42 %) represented these types of microbes [11]. Similarly, previous observational studies reported rates of polymicrobial etiology of VAP that ranged between 28 % and 50 % when PPMs and non-pathogenic microbes were analyszed [34–37]. Having included non-pathogenic microbes would have increased the rate of polymicrobial pneumonia to 30 % in our population. Conversely, we report in our study that 16 % of patients with ICUAP had polymicrobial etiology when we restricted the analysis to PPMs. A previous study that used the same criteria reported a similar rate (10 %) for VAP of polymicrobial etiology [38].

A relevant issue in the polymicrobial etiology of ICUAP is the potential prognostic implications. For that reason, finding predictors of polymicrobial pneumonia could hypothetically be of potential interest. However, in a clinical setting of highly appropriate initial antibiotic treatment, as reported in the present study for both groups, all the outcomes, including length of stay, ventilator-free days and mortality, were similar in the two groups. Indeed, in our study the numbers of patients with MDR or XDR pathogens in our study did not differ with pneumonia of polymicrobial etiology. In addition, when patients were clustered into non-ventilated ICUAP and VAP, there were also no statistically significant differences in these outcomes.

In our study, the only variables associated with polymicrobial etiology were absence of chronic heart disease and prior hospital admission, and the presence of pleural effusion, which was twice as high in the polymicrobial group. We have previously reported that patients with clinical diagnosis of both community-acquired pneumonia and ICUAP and negative microbiology more frequently have chronic heart disease [14, 39]. Both studies suggested that some of these cases might also represent, at least in part, fluid overload due to congestive heart failure added to the underlying inflammatory process potentially mimicking pneumonia. Similarly, underlying chronic heart disease might hypothetically have contributed to the development of pulmonary congestion in patients with presumably lower bacterial burden, such as those with pneumonia of monomicrobial etiology.

We do not have a clear explanation for the association between a higher rate of pleural effusion and polymicrobial pneumonia. The isolation of MSSA in pleural fluid culture from two patients was not sufficiently decisive to allocate them to the polymicrobial pneumonia group; in one patient, this pathogen was concomitantly identified in blood and tracheal aspirate cultures, while in the other, both *H. influenzae* and *Klebsiella* spp were isolated in a tracheal aspirate culture. The association between pleural effusion and ICUAP of polymicrobial etiology needs to be confirmed in future prospective studies.

Neither prior antibiotic treatment nor late-onset pneumonia, as in the previous French study [11], were predictors of polymicrobial pneumonia in our study. However, prior antibiotic treatment was associated with the presence of MDR or XDR pathogens in our study. In addition we found no association between the severity of presentation and polymicrobial etiology. This finding complements a previous study in 343 patients with ICUAP that concluded that severity of illness seems not to affect etiology [9].

We also found no differences in the serum levels of any inflammatory biomarkers that we measured at the onset of pneumonia. In relation to the etiology of ICUAP, we have recently reported that inflammatory biomarkers were unable to differentiate between patients with positive and negative microbiology [14], or patients with or without MDR pathogens [9]. All these studies confirm that inflammatory biomarkers are not useful in predicting the etiology of ICUAP.

The main strengths of our study are the prospective design, the detailed description of microbiology in ICUAP, the inclusion of both VAP and non-ventilator ICUAP, the exclusion of non-potentially pathogenic microorganisms from the analysis, the assessment of several inflammatory biomarkers, and the follow up of patients up to 90 days. However, in our study we excluded immunosuppressed patients. Consequently, we cannot extrapolate our results to this population.

This study has some limitations. First, it was conducted in a single center, so the extrapolation of these findings to other settings must be done cautiously. Second, we did not use molecular microbiological techniques that are potentially more sensitive. However, the experience of using these techniques in ICUAP, and in non-ventilator ICUAP and VAP, is still scarce. Third, the sample size of our study is not enough large to make a robust analysis of all related questions; when comparing several characteristics between the two groups of patients the current study was underpowered. It is important to outline that this is a non-interventional study and our purpose was only to describe clinical findings. Fourth, there were no adjustments made for multiple comparisons.

## Conclusion

The etiology of ICUAP with microbiologic confirmation was polymicrobial in 16 % of patients. Pleural effusion and absence of chronic heart disease are associated with polymicrobial pneumonia. When empiric antibiotic treatment is frequently appropriate, polymicrobial etiology does not influence the outcome of ICUAP.

## Key messages

- Polymicrobial etiology of ICU-acquired pneumonia accounted for 16 % cases with microbiologic confirmation
- Polymicrobial etiology of ICU-acquired pneumonia was associated with pleural effusion and absence of chronic heart disease
- Polymicrobial etiology did not result in higher incidence of multi-drug- and extensive-drug-resistant pathogens
- When empiric treatment is appropriate, polymicrobial etiology does not influence the outcome of ICU-acquired pneumonia

## Abbreviations

APACHE: acute physiology and chronic health evaluation
ARDS: acute respiratory distress syndrome
BAL: bronchoalveolar lavage
CPIS: clinical pulmonary infectious score
ICU: intensive care unit
ICUAP: ICU-acquired pneumonia
IL: interleukin
MDR: multi-drug-resistant
MRSA: methicillin-resistant *Staphylococcus aureus*
MSSA: methicillin-sensitive *Staphylococcus aureus*
PaO_2_/FiO_2_: arterial partial pressure of oxygen/inspired oxygen fraction
PDR: pan-drug resistance
PPM: potentially pathogenic microorganism
SAPS: simplified acute physiology score
SOFA: sepsis-related organ failure assessment
TBAS: tracheobronchial aspirates
TNF: tumor necrosis factor
VAP: ventilator-associated pneumonia
XDR: extensive-drug-resistant
